# Predicting of tunneling resistivity between adjacent nanosheets in graphene–polymer systems

**DOI:** 10.1038/s41598-023-39414-w

**Published:** 2023-08-01

**Authors:** Yasser Zare, Nima Gharib, Dong-Hyun Nam, Young-Wook Chang

**Affiliations:** 1 Biomaterials and Tissue Engineering Research Group, Department of Interdisciplinary Technologies, Breast Cancer Research Center, Motamed Cancer Institute, ACECR, Tehran, Iran; 2 College of Engineering and Technology, American University of the Middle East, Egaila, 54200 Kuwait; 3 Department of Materials Science and Chemical Engineering, BK21 FOUR ERICA-ACE Center, Hanyang University ERICA, Ansan, 15588 Korea

**Keywords:** Engineering, Materials science

## Abstract

In this work, the tunneling resistivity between neighboring nanosheets in grapheme–polymer nanocomposites is expressed by a simple equation as a function of the characteristics of graphene and tunnels. This expression is obtained by connecting two advanced models for the conductivity of graphene-filled materials reflecting tunneling role and interphase area. The predictions of the applied models are linked to the tested data of several samples. The impressions of all factors on the tunneling resistivity are evaluated and interpreted using the suggested equation. The calculations of tunneling resistivity for the studied examples by the model and suggested equation demonstrate the same levels, which confirm the presented methodology. The results indicate that the tunneling resistivity decreases by super-conductive graphene, small tunneling width, numerous contacts among nanosheets and short tunneling length.

## Introduction

Graphene-filled products can be utilized in different grounds like electronics, electromagnetic shielding, sensing, energy devices and diodes, because graphene displays ideal electrical, mechanical, thermal and chemical properties^1–18^. The higher aspect ratio and bigger surface area of graphene nanosheets compared to CNT cause lower percolation inception and more conductivity^19^. So, the investigators have widely focused on the polymer graphene nanocomposites to optimize their performances. Most recent studies on polymer graphene nanocomposites have tried to prepare the samples with little percolation inception and great conductivity by low filler amount^20–22^. The percolation inception inversely connects to the aspect ratio of nanofiller as the ratio of diameter to thickness^23, 24^. Therefore, many parameters such as the dimensions, dispersion quality and aggregation/agglomeration of nanoparticles handle the percolation inception and thus, the nanocomposite’s conductivity.

Some new parameters attributed to nanoscale including tunneling effect and interphase can also govern the percolation inception. The tunneling tool mainly controls the conductivity of nanocomposites (abbreviated as conductivity here), since the electrons can be easily transported over small tunnels between neighboring particles^25–28^. In fact, the conductivity does not need the physical joining of nanoparticles and so, the tunneling effect changes the percolation inception in nanocomposites. However, only few investigators have concentrated on the tunneling conductivity in CNT-based products^29–31^. Moreover, the interphase, owing to the large external area of nanoparticles can efficiently decrease the percolation inception. The interphase is the depleted polymer layer at the filler–polymer interface^32, 33^. The interphase areas covering the nanoparticles can join together and construct the nets in the samples^34–38^. This attractive topic has been studied for mechanical behavior of polymer nanocomposites^39–43^, but the role of interphase in the conductivity was negligibly studied.

Various equations were advanced for the conductivity of CNT-filled examples assuming the factors for CNT such as amount, waviness, conduction and aspect ratio^44–47^. Also, few studies have reported the significances of tunneling effect and interphase on the conductivity of CNT products^34, 47, 48^. However, the modeling works on the conductivity of graphene-based systems are really incomplete. The former works usually correlated the percolation inception to filler aspect ratio and judged the conductivity by conventional power-law equation^49–51^. In summary, the foregoing studies have not considered the interphase and tunnels in the percolation inception and conductivity, while these factors mainly control the mentioned terms.

In this study, two models for the conductivity of graphene-based samples are coupled to describe the tunnel resistivity by the characteristics of graphene and tunnels. The predictability of the applied models is weighed by the experimented conductivity of some samples from literature. Furthermore, the novel model and the submitted equation are used to get the tunnel resistivity. Additionally, the novel equation is utilized to evaluate and validate the effects of different factors on the tunnel resistivity.

## Theoretical remarks

**Figure 1:**
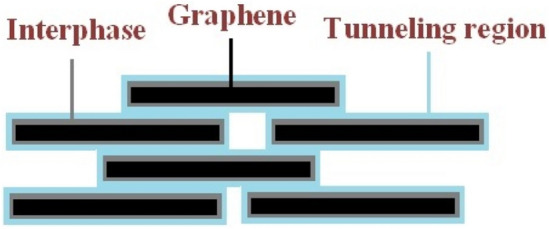
The components in polymer graphene nanocomposites.

A polymer nanocomposite includes the nanofiller, surrounding interphase and tunnels between neighboring nanoparticles. Figure 1 displays the mentioned components in a graphene nanocomposite in which the interphase covers the nanosheets and the tunneling zones form between nearby nanosheets.

The conductivity in polymer nanocomposites needs the networking of conductive nanofiller, which occurs above an essential filler amount as percolation inception^20, 52^.

The percolation inception in polymer graphite nanocomposites was formulized ^24^ by:

1 $$\varphi_{p} = \frac{{27\pi D^{2} t}}{{4(D + \lambda )^{3} }}$$

where “D” and “t” show the diameter and thickness of sheets and “λ” is tunneling length. Equation 1 was derived for graphite nanoparticles with high aspect ratio (D/t). Since both graphene and graphite have high aspect ratio, we used Eq. (1) for graphene-filled samples. Actually, very thin and long platelets (high aspect ratio) are similar to graphene nanosheets with high aspect ratio. Both thickness and diameter of platelets/nanosheets play the main role in the percolation onset, as mentioned in Eq. (1).

Assuming D >  > λ, Eq. (1) is simplified as:

2 $$\varphi_{p} = \frac{27\pi t}{{4D}}$$

The latter equation can be advanced assuming tunneling and interphase zones as:

3 $$\varphi_{p} = \frac{{27\pi t^{2} }}{{4Dt + 2(Dt_{i} + D\lambda )}}$$

where “t_i_” is interphase deepness. This equation represents that the percolation inception links to filler size, interphase deepness and tunneling length. Equation 3 is true, because the percolation onset adversely depends on the filler aspect ratio^23, 24^ and also this equation suggests a dimensionless parameter. Equation 3 is valid for only graphene-filled samples and cannot predict the percolation if the shape of the fillers is different.

The interphase areas also grow the efficiency of nanofiller in nanocomposites, because both nanofiller and interphase produce the networks. The total volume fraction of interphase in polymer graphene nanocomposites^2^ is given by:

4 $$\varphi_{i} = \varphi_{f} \left( {\frac{{2t_{i} }}{t}} \right)$$

where “$\varphi_{f}$” is filler volume share. The interphase areas can add to the filler nets; so, the effective volume fraction of nanofiller includes the fractions of graphene and surrounding interphase as:

5 $$\varphi_{eff} = \varphi_{f} + \varphi_{i} = \varphi_{f} \left( {1 + \frac{{2t_{i} }}{t}} \right)$$

Also, the percentage of nanosheets contributing to the conductive networks^53^ can be estimated by:

6 $$f = \frac{{\varphi_{f}^{1/3} - \varphi_{p}^{1/3} }}{{1 - \varphi_{p}^{1/3} }}$$

Assuming “$\varphi_{eff}$” (Eq. 5) develops the “f” to:

7 $$f = \frac{{\varphi_{eff}^{1/3} - \varphi_{p}^{1/3} }}{{1 - \varphi_{p}^{1/3} }}$$

expressing that both graphene and interphase parts affect the dimensions of conductive networks.

To consider the role of tunnels between nanosheets in the conductivity, the extended nanosheets are suggested. In other words, an extended nanosheet contains the graphene and tunneling zones.

The total intrinsic resistance of a prolonged nanosheet is proposed by:

8 $$R_{ext} = R_{f} + R_{c}$$

where “R_f_” and “R_c_” are the basic resistances of graphene and tunnels, in that order.

“R_f_” is expressed as:

9 $$R_{f} = \frac{1}{{t\sigma_{f} }}$$

where “σ_f_” is the conduction of graphene.

The tunnel resistance also consists of the resistances of graphene and insulated polymer within the tunnels. Accordingly, the tunnel resistance is stated by the resistances of graphene (R_g_) and polymer (R_t_) in the contact zones as:

10 $$R_{c} = R_{g} + R_{t}$$

“R_g_” and “R_p_” can be suggested^54^ by:

11 $$R_{g} = \frac{1}{{\sigma_{f} d}}$$

12 $$R_{t} = \frac{\rho \lambda }{S} = \frac{\rho \lambda }{{d^{2} }}$$

where “d” is tunnel diameter, “ρ” is tunnel resistivity by insulated polymer and “S” shows tunnel area (S ≈ d^2^).

Replacing of Eqs. (11) and (12) into Eq. (10) results in the intrinsic resistance of tunnels as:

13 $$R_{c} = \frac{1}{{\sigma_{f} d}} + \frac{\rho \lambda }{{d^{2} }}$$

which offers the whole intrinsic resistance of prolonged nanosheets (Eq. 8) as:

14 $$R_{ext} = \frac{1}{{t\sigma_{f} }} + \frac{1}{{\sigma_{f} d}} + \frac{\rho \lambda }{{d^{2} }} \,$$

The total resistance of prolonged nanosheets assuming tunnels is applied to foresee the conductivity.

The total conductivity of extended nanosheets (S/m) can be expressed by reversing “R_ext_” as:

15 $$\sigma_{ext} = \frac{1}{tR} = \frac{1}{{\frac{1}{{\sigma_{f} }} + \frac{t}{{\sigma_{f} d}} + \frac{t\rho \lambda }{{d^{2} }}}} \,$$

Two researchers^45^ obtained an equation for conductivity (random CNT) as:

16 $$\sigma = \sigma_{0} + \frac{{f\varphi_{f} \sigma_{f} }}{3}$$

where “σ_0_” is the conductivity of polymer medium (10^−13^–10^−15^ S/m), which can be unnoticed. Equation (16) can be applied to guesstimate the conductivity in graphene-filled samples.

When the operative filler amount (Eq. 5) and the conductivity of prolonged sheets (Eq. 15) are reflected in Eq. (16), the conductivity is given by:

17 $$\sigma = \frac{{f\varphi_{eff} \sigma_{ext} }}{3} = \frac{{f\varphi_{eff} }}{{3\left( {\frac{1}{{\sigma_{f} }} + \frac{t}{{\sigma_{f} d}} + \frac{t\rho \lambda }{{d^{2} }}} \right)}}$$

which correlates the conductivity to graphene, interphase and tunnel properties.

Weber and Kamal^55^ also formulated the longitudinal resistivity for fiber-based composites by:

18 $$\rho_{l} = \frac{{A_{f} \rho_{f} X}}{{f\varphi_{f} dl\cos^{2} \theta }}$$

where “A_f_” shows filler cross-section zone, “l” is fiber length, “ρ_f_” is the resistivity of fiber and “θ” is angle among fibers and current path. In addition, “X” is links to contact number (m) by:

19 $$X = \frac{1}{0.59 + 0.15m}$$

where the highest “m” is 15^55^.

The composite’s conductivity can be gotten by inverse “ρ_l_” as:

20 $$\sigma = \frac{{f\varphi_{f} dl\cos^{2} \theta }}{{A_{f} \rho_{f} X}}$$

which can be advanced for the graphene systems.

The cross-section zone of graphene can be obtained as:

21 $$A_{f} = tD$$

Also, “l” is replaced by “D” and σ_f_ = 1/ρ_f_. Additionally, for 3-D haphazard spreading of nanoparticles in the samples^56^, it is considered that:

22 $$\cos^{2} \theta = \frac{1}{3}$$

So, Eq. (20) can be expressed for graphene systems by operative filler amount (Eq. 5) as:

23 $$\sigma = \frac{{f\varphi_{eff} d\sigma_{f} }}{3tX}$$

Moreover, the tunneling length mainly impresses the conductivity, since it manages the electron transferring at tunnels (Fig. 1). The tunneling length (λ) correlates to $\varphi_{f}^{ - 1/3}$^53, 57^. Since Eq. (20) suggests a linear link amid conductivity and “$\varphi_{f}^{{}}$”, the conductivity connects to “λ^−3^”.

Based on these remarks, the latter equation can consider the tunneling length as:

24 $$\sigma = \frac{{f\varphi_{eff} d\sigma_{f} }}{{3tX\left( {\frac{\lambda }{z}} \right)^{3} }}$$

where “z” is tunneling characteristics length, which is considered as 0.1 nm for graphene-filled samples. This equation signifies a model for conductivity of products by graphene dimensions, graphene conduction, number of contacts, graphene amount in the nets, tunnel diameter, interphase deepness and tunneling length.

Now, Eqs. (17) and (24) can be combined to express an equation for tunnel resistivity (ρ).

Equations (17) and (24) are coupled as:

25 $$\frac{{f\varphi_{eff} }}{{3\left( {\frac{1}{{\sigma_{f} }} + \frac{t}{{\sigma_{f} d}} + \frac{t\rho \lambda }{{d^{2} }}} \right)}} = \frac{{f\varphi_{eff} d\sigma_{f} }}{{3tX\left( {\frac{\lambda }{z}} \right)^{3} }}$$

which expresses the tunnel resistivity as:

26 $$\rho = \frac{{dtX\left( {\frac{\lambda }{z}} \right)^{3} - d^{2} - td}}{{t\sigma_{f} \lambda }}$$

According to this equation, the tunnel resistivity in polymer graphene systems associates to the features of graphene and tunnels. This equation clearly demonstrates the significances of each parameter on the tunnel resistivity.

## Results and discussion

### Application of equations

The predictability of both models is confirmed by the experimented levels of examples from previous articles. Also, the tunnel resistivity for the samples is calculated and compared. Four graphene examples including polyimide (PI) ($\varphi_{p}^{{}}$ = 0.0015, D ≈ 5 μm, t = 3 nm)^58^, polystyrene (PS) ($\varphi_{p}^{{}}$ = 0.001, D ≈ 2 μm, t = 1 nm)^59^ (No. 1), poly (ethylene terephthalate) (PET) ($\varphi_{p}^{{}}$ = 0.005, D ≈ 2 μm, t = 2 nm)^60^ and PS ($\varphi_{p}^{{}}$ = 0.0005, D ≈ 4 μm, t = 1 nm)^61^ (No. 2) were chosen from earlier researches. The values o f (t_i_, λ) can be analyzed by fitting the percolation inception to Eq. (3). Using this equation, (t_i_, λ) levels of (7, 9), (10, 9), (3, 4) and (7, 10) nm are obtained for PI, PS (No. 1), PET and PS (No. 2) nanocomposites, correspondingly. These calculations reveal the foundation of unlike interphase and tunnels in the nanocomposites. The densest interphase and the largest tunnels are shown in PS/graphene (No. 1) and PS/graphene (No. 2) samples, while the least levels of interphase deepness and tunneling length are observed in PET/graphene sample. It is obvious that a deep interphase and a big tunnel decrease the percolation inception. So, assumption of interphase deepness and tunneling length in the percolation inception is necessary, because these parameters largely manipulate the percolation inception in nanocomposites. Using these calculations, it is possible to calculate the conductivity by the developed models. Graphene conductivity and cos^2^ (θ) are reflected as 10^5^ S/m and 1/3, in that order.

**Figure 2:**
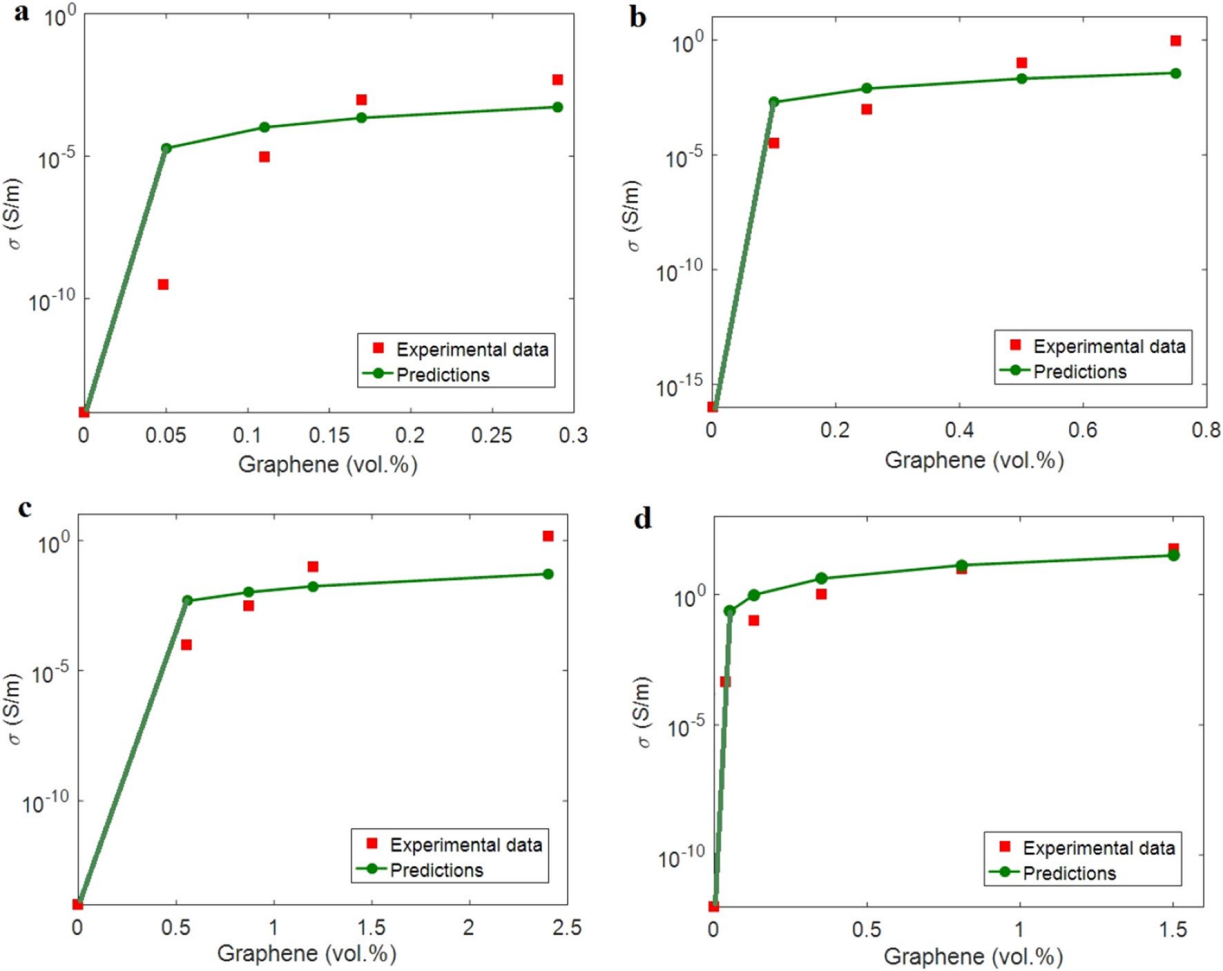
The experimented conductivity and the calculations by Eqs. (17) and (24) for (**a**) PI^58^, (**b**) PS^59^ (No. 1), (**c**) PET^60^ and (**d**) PS (No. 2)^61^ graphene products.

Figure 2 reveals the experimented and theoretical ranks of conductivity for the examples. As observed, the calculations demonstrate fine matching with the experimented data of examples. As a result, both models suitably visualize the conductivity for the samples, which confirm their predictability for all polymer graphene nanocomposites. The values of (m, d) (d in nm) by Eq. (24) are obtained as (5, 10), (3, 10), (4, 5) and (120, 400) for PI, PS (No. 1), PET and PS (No. 2) nanocomposites, in that order. These results display the different ranges of contact number and tunnel diameter in the nanocomposites. The most desirable levels of these parameters are obtained for PS/graphene (No. 2) sample. Conferring to the experimented results in Fig. 2, this sample displays the most level of conductivity. So, it is concluded that the number of contacts and the tunnel diameter mainly affect the conductivity. Also, Eq. (17) calculates the tunnel resistivity (ρ) as 6.04, 7.8, 0.67 and 21.4 Ω.m for PI, PS (No. 1), PET and PS (No. 2) examples, correspondingly. The utmost and the lowermost levels of tunnel resistivity are shown in PS/graphene (No. 1) and PET/graphene nanocomposites, respectively. The same values of tunnel resistivity are also obtained for the reported samples using the suggested equation (Eq. 26). In fact, both Eqs. (17) and (26) present the same levels for tunnel resistivity based on the experimental results of conductivity and percolation inception. These outputs support the new equation for tunnel resistivity in nanocomposites. In other words, Eq. (26) can be used to foresee the tunnel resistivity.

### Analysis of parameters

In this section, the inspirations of all factors on the tunnel resistivity are evaluated using the novel equation (Eq. 26).

**Figure 3:**
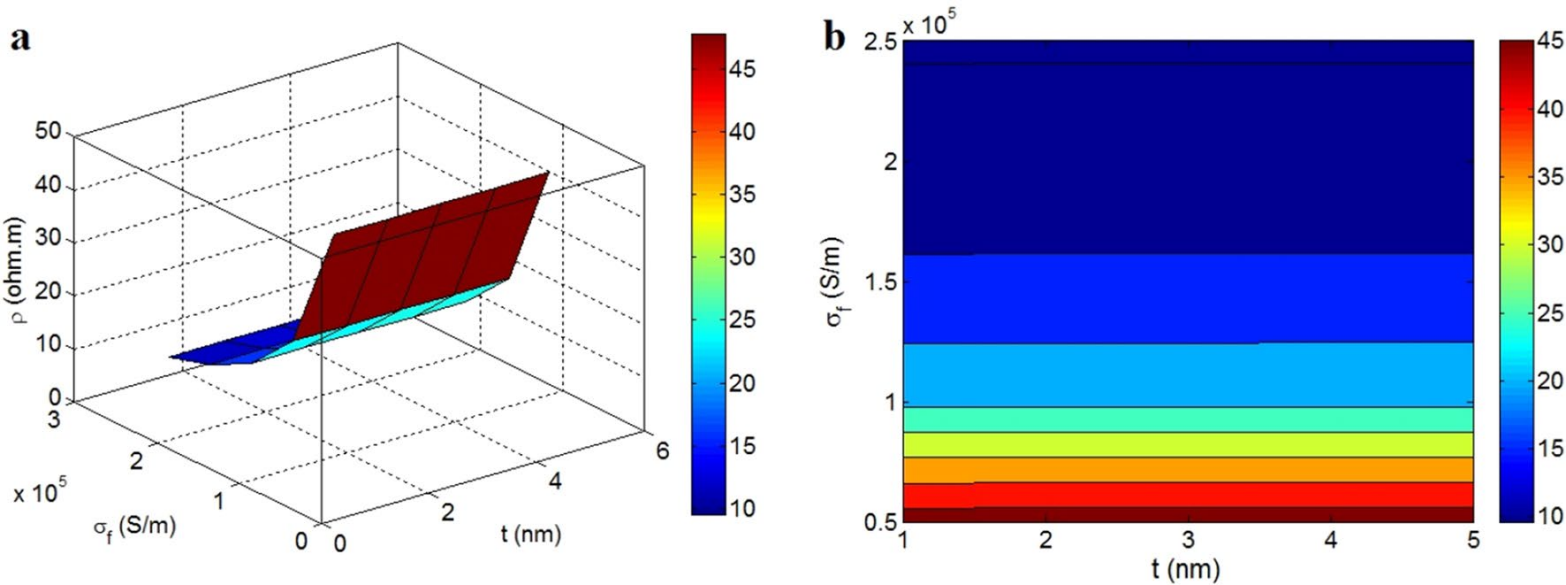
The impressions of “t” and “σ_f_” on the tunnel resistivity (Eq. 26) by (**a**) 3-D and (**b**) contour designs.

Figure 3 illustrates the roles of “t” and “σ_f_” in the tunnel resistivity at λ = 5 nm, m = 10, d = 200 nm and z = 0.1 nm. The lowest tunnel resistivity as 10 Ω.m is observed at σ_f_ > 1.65*10^5^ S/m, but the tunnel resistivity increases to 45 Ω.m at σ_f_ = 0.5*10^5^ S/m. As a result, the graphene conduction inversely influences the tunnel resistivity, while the thickness of graphene nanosheets cannot affect it. In fact, a super-conductive nanofiller can mainly decrease the tunnel resistivity in nanocomposites, which promotes the conductivity. However, the graphene thickness is an ineffective factor, which cannot change the tunnel resistivity.

The graphene nanosheets cover the tunnels in the nanocomposites. Undoubtedly, the conductive graphene significantly decreases the tunnel resistivity, because it can facilitate the transference of electrons through tunnels. On the other hand, poor-conductive graphene cannot reduce the tunnel resistivity, because it cannot affect the resistance of tunnels containing insulated polymer film and graphene nanosheets. So, it is meaningful to observe an inverse relation between tunnel resistivity and graphene conduction. Additionally, the thickness of graphene cannot control the tunnel resistivity, because its role in the tunnels is negligible. Conclusively, the size of graphene nanosheets cannot affect the tunnel resistivity, but the conduction of graphene positively decreases it.

**Figure 4:**
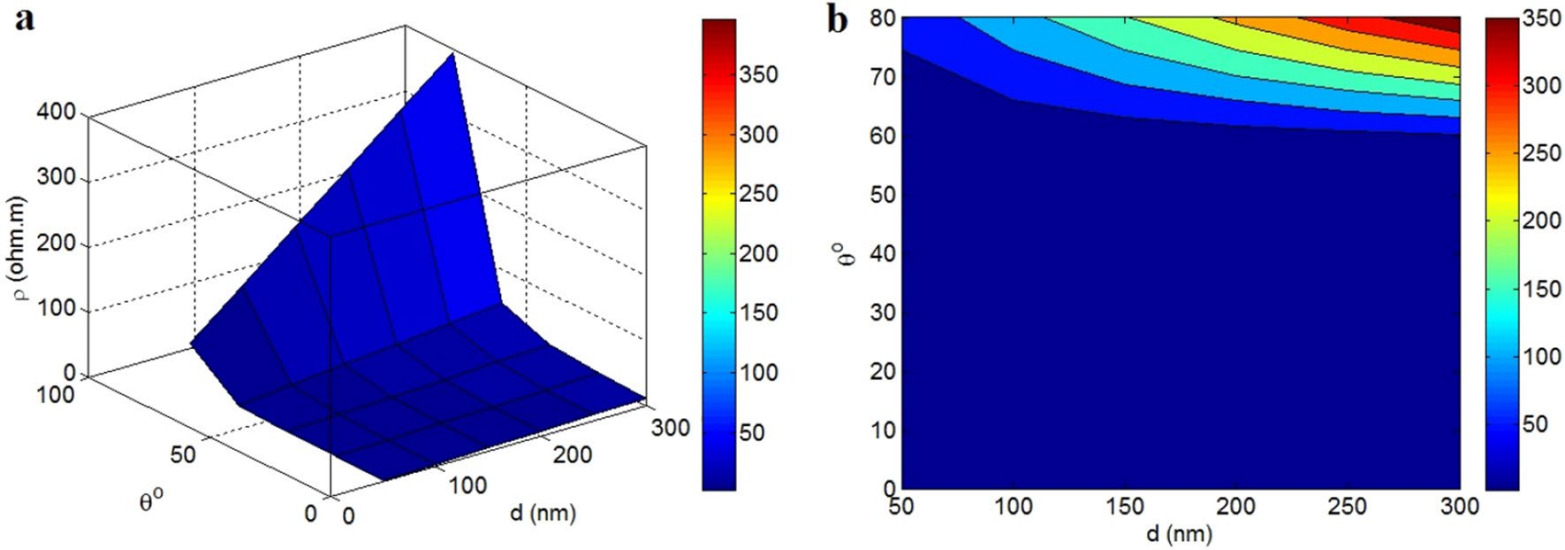
Expression of tunnel resistivity (Eq. 26) by “d” and “θ”: (**a**) 3-D and (**b**) contour schemes.

Figure 4 also shows the dependency of tunnel resistivity on “d” and “θ” at t = 2 nm, λ = 5 nm, σ_f_ = 10^5^ S/m, m = 10 and z = 0.1 nm. The smallest level of tunnel resistivity as about 0 is obtained by θ < 60°, while the highest tunnel resistivity is calculated at the highest ranges of both “d” and “θ”. The calculations demonstrate that the maximum tunnel resistivity of 350 Ω.m is detected at d = 300 nm and θ = 80°. Consequently, the diameter of contact area between adjacent nanosheets and the filler angle directly change the tunnel resistivity. In fact, it is important to decrease the levels of tunnel diameter and filler angle to obtain a poor tunnel resistivity.

The tunnel diameter between nanosheets determines the extent of tunnels, because two neighboring nanosheets form the tunneling space. In other words, a big tunnel diameter causes a large tunnel in the nanocomposites, whereas less “d” forms a small tunneling zone. Since the tunnels mainly contains the insulated polymer matrix, a large tunnels produces a high tunnel resistivity. On the other hand, the less value of tunnel diameter between adjacent nanosheets yields a small tunnel. In this condition, the tunnel resistivity mainly decreases, because of the small tunnels. Moreover, the nanosheets oriented to the current direction can easily and effectively transfer the charges and improve the conductivity, while a high angle between nanosheets and electron current limits the tunneling conductivity. Accordingly, a low “θ” significantly increases the electron transportation and decreases the tunnel resistivity. However, a high “θ” produces an improper implementation of nanosheets in the nanocomposite, which grows the tunnel resistivity. Therefore, both tunnel diameter and orientation angle logically influence the tunnel resistivity in nanocomposites.

**Figure 5:**
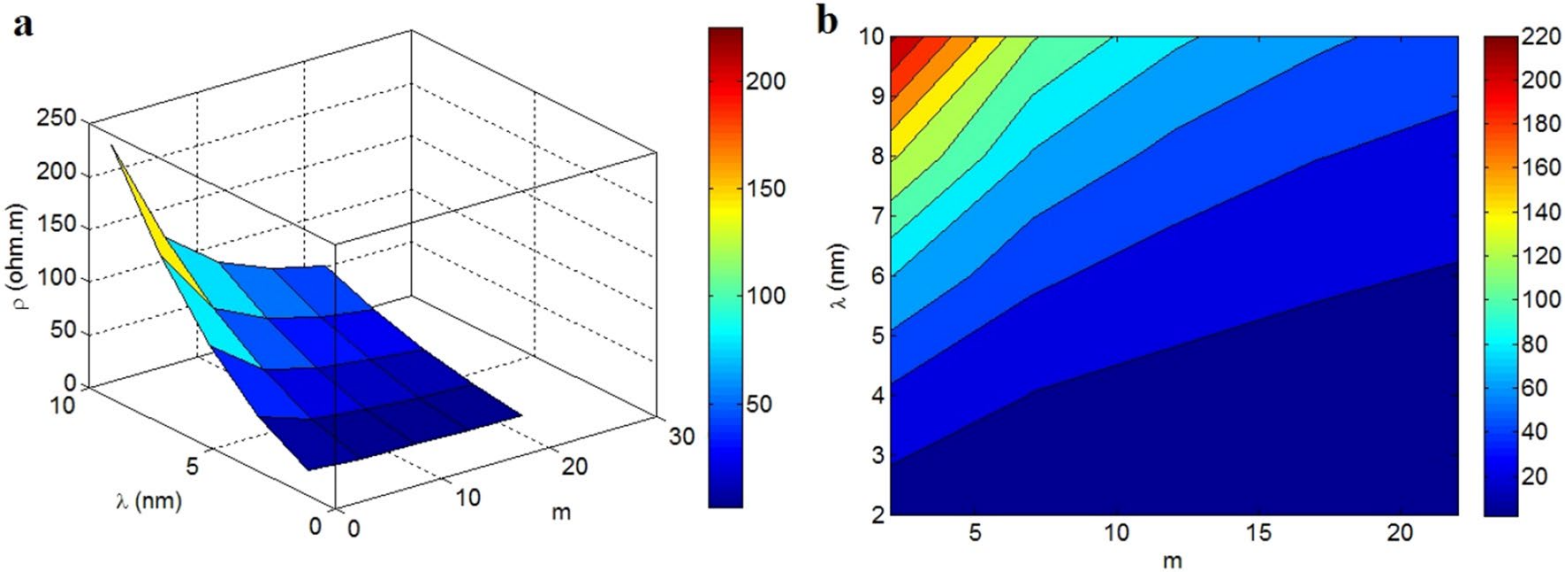
(**a**) 3-D and (**b**) contour designs for the variation of tunnel resistivity (Eq. 26) at altered series of “m” and “λ”.

The stimuli of “m” and “λ” on the tunnel resistivity are observed in Fig. 5. The uppermost tunnel resistivity of 220 Ω.m is obtained in m = 2 and λ = 10 nm, but the tunnel resistivity largely declines to 0 at λ < 3 nm or m > 15 and λ < 5 nm. Accordingly, abundant contacts among sheets and a minor tunneling length attain a deprived tunnel resistivity. In contrast, a less quantity of contacts and long tunnel negatively raise the tunnel resistivity.

A high number of contacts reduce the tunnel resistivity, but the limited ranges of contact number increase it. In fact, the large links between conductive graphene nanosheets produce the conductive tunneling zones in nanocomposites weakening the tunnel resistivity. However, the low extent of contacts demonstrates the presence of insulated polymer matrix between nanosheets, which considerably increases the tunnel resistivity. As a result, the suggested equation (Eq. 26) properly predicts the correlation between tunnel resistivity and contact number. In addition, a high tunneling length between nanosheets shows the existence of a thick polymer layer in the tunnels. Since the insulated polymer matrix weakens the transportation of electrons in the tunnels, it is reasonable to obtain a high tunnel resistivity in this condition, due to the high amount of insulated material. Nevertheless, a small tunneling length displays a thin polymer film between neighboring nanosheets, which causes a poor tunnel resistivity. The former articles reported that the large tunnel decreases the tunneling conductivity, due to the restricted electron shifting over tunnels or high tunnel resistivity^2, 62^. Based on these reasons, the suggested equation reasonably indicates the role of tunneling length in the tunnel resistivity.

## Conclusions

The tunnel resistivity in polymer graphene nanocomposites was defined as a function of the characteristics of graphene and tunnels. This equation was extracted by extending the graphene nanosheets and upgrading a conventional model. The estimates of these models demonstrate fine arrangements with the experimented data. Also, the tunnel resistivity of the reported samples calculated by the developed model and the suggested equation give similar values, which approve the presented equations. σ_f_ > 1.65*10^5^ S/m produces the tunnel resistivity of 10 Ω.m, but the tunnel resistivity grows to 45 Ω.m at σ_f_ = 0.5*10^5^ S/m. Consequently, the graphene conduction inversely handles the tunnel resistivity, nonetheless the thickness of graphene nanosheets cannot affect it. The smallest level of tunnel resistivity as about 0 is also obtained by θ < 60°, while the highest tunnel resistivity is calculated by the highest ranges of both “d” and “θ”. As a result, the diameter of contact area between nanosheets and the filler angle directly govern the tunnel resistivity. In addition, the highest tunnel resistivity of 220 Ω.m is gotten by m = 2 and λ = 10 nm, nevertheless the tunnel resistivity mostly declines to around 0 at λ < 3 nm or m > 15 and λ < 5 nm. Therefore, plentiful contacts among nanosheets and a small tunneling length achieve a low tunnel resistivity in nanocomposites.

## Data Availability

The data that support the findings of this study are available on request from corresponding author.
